# RNA polymerase III transcription in cancer: the BRF2 connection

**DOI:** 10.1186/1476-4598-10-47

**Published:** 2011-04-25

**Authors:** Stephanie Cabarcas, Laura Schramm

**Affiliations:** 1 National Cancer Institute, Laboratory of Cancer Prevention, Cancer Stem Cell Section, 1050 Boyles Street, Building 560, Room 21-81, Frederick, MD 21702, USA; 2 Department of Biological Sciences, St. John's University, Queens, New York 11439, USA

## Abstract

RNA polymerase (pol) III transcription is responsible for the transcription of small, untranslated RNAs involved in fundamental metabolic processes such mRNA processing (U6 snRNA) and translation (tRNAs). RNA pol III transcription contributes to the regulation of the biosynthetic capacity of a cell and a direct link exists between cancer cell proliferation and deregulation of RNA pol III transcription. Accurate transcription by RNA pol III requires TFIIIB, a known target of regulation by oncogenes and tumor suppressors. There have been significant advances in our understanding of how TFIIIB-mediated transcription is deregulated in a variety of cancers. Recently, BRF2, a component of TFIIIB required for gene external RNA pol III transcription, was identified as an oncogene in squamous cell carcinomas of the lung through integrative genomic analysis. In this review, we focus on recent advances demonstrating how BRF2-TFIIIB mediated transcription is regulated by tumor suppressors and oncogenes. Additionally, we present novel data further confirming the role of BRF2 as an oncogene, extracted from the Oncomine database, a cancer microarray database containing datasets derived from patient samples, providing evidence that BRF2 has the potential to be used as a biomarker for patients at risk for metastasis. This data further supports the idea that BRF2 may serve as a potential therapeutic target in a variety of cancers.

## Introduction

Cancer is a major health problem afflicting millions of Americans annually and despite tremendous research and treatment advances, is still the leading cause of death amongst men and women younger than age 85 years [1]. A dominant characteristic of many types of cancer cells is its ability to proliferate uncontrollably. RNA polymerase (pol) III contains the largest number of subunits (17 subunits) and is responsible for the transcription of small, less than 300 nucleotides, untranslated RNAs involved in fundamental metabolic processes, such as RNA processing (U6 snRNA) and translation (tRNAs), which contribute to cell proliferation [2]. Thus, deregulation of RNA pol III transcription can lead to aberrant production of critical RNAs contributing to uncontrolled cell growth, a hallmark trait of many types of cancer.

**Figure 1:**
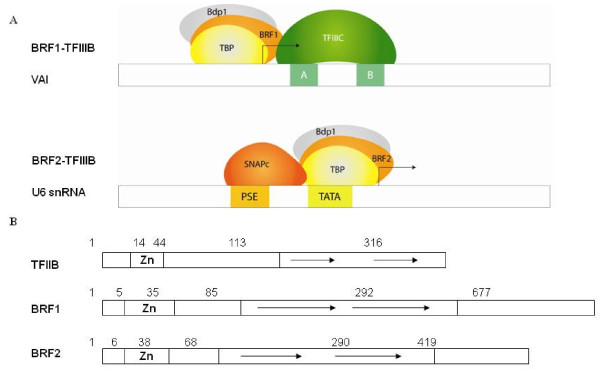
**Gene internal and external TFIIIB**. (A) Schematic of gene-internal TFIIIB (BRF1-TFIIIB) and gene-external human TFIIIB (BRF2-TFIIIB), note the difference in complexes is BRF1 and BRF2. (B) Schematic representation of TFIIB, BRF1 and BRF2 protein structures. Note the unrelated C-terminal extensions in the TFIIIB subunits BRF1 and BRF2. (Adapted from [2]).

Like all eukaryotic polymerases, RNA pol III cannot recognize its target promoters directly and accurate initiation requires TFIIIB [2-4]. In higher eukaryotes, thus far, two forms of TFIIIB have been identified [2-4]. BRF1-TFIIIB required for transcription by gene internal RNA pol III promoters (tRNA) contains Bdp1, TBP and BRF1 (Figure 1). BRF2-TFIIIB required for transcription from RNA pol III gene external promoters contain Bdp1, TBP and BRF2 (Figure 1) [2]. Examples of genes transcribed by BRF2-TFIIIB include the human U6 snRNA gene involved in RNA splicing, the 7SK gene whose RNA product has been demonstrated to negatively regulate RNA Pol II transcription elongation by binding to the elongation factor P-TEFb, the RNase mitochondrial RNA processing (MRP) which participates in pre-rRNA processing, novel noncoding RNAs of unknown function (reviewed in [2,5]).

BRF2 (TFIIB-related factor 2) shares structural features with TFIIB and BRF1 (Figure 1B). TFIIB, BRF1 and BRF2 all contain N-terminal zinc ribbon domains, core domains containing imperfect repeats; BRF1 and BRF2 have unrelated C-terminal extensions (Figure 1B) [2]. The C-terminus of BRF2 is required for association with TBP and SNAPc (small nuclear activating protein complex) on the U6 promoter [6].

## RNA pol III and cancer

Many different transformed cell types have been shown to have increased products of RNA pol III, when transformed by DNA tumor viruses, as well as chemical carcinogens [7-11] and their relevance has been validated in tumors of the breast, cervix, esophagus, lung, ovary, parotid, and tongue, but not in corresponding normal tissues tumors [12]. Specifically, RT-PCR analysis has demonstrated that tRNAs are overproduced consistently in human ovarian cancers [13]. Also, tRNA levels have been shown to be 10-fold higher in breast cancer cells than in normal cells [14]. These increases are not simply a consequence of rapid cell proliferation in cancer [15], but instead contribute to tumorigenesis, as it has been demonstrated that overexpression of tRNAi^Met ^induces proliferation and immortalization of fibroblasts [16].

Activation of TFIIIB activity has been noted in a variety of cancers types. Increased TBP expression has been observed in a clinically significant number of human colon cancers [17]. Also, overexpression of BRF1 has also been shown to transform mouse embryo fibroblasts [16]. Bdp1 is overexpressed in cells transformed by papovaviruses [11], but changes in expression levels in specific human cancer types have not been determined. Amplification of BRF2 has been noted in breast cancer [18,19] and more recently a human bladder cancer cell line [20]. Recently, Lockwood et al. demonstrated that genetic activation of BRF2 represents a unique mechanism of squamous cell carcinoma tumorigenesis, also providing the first clinical evidence implicating BRF2 as a novel lineage-specific oncogene in lung squamous cell carcinoma [20]. This review will focus on BRF2-TFIIIB activity in cancer.

## Regulation of BRF2-TFIIIB activity by oncogenes and tumor suppressors

RNA pol III transcription is tightly regulated during the cell cycle to ensure normal cellular growth [21]. Cellular levels of RNA pol III are specifically increased in tissues isolated from mice with myeloma compared to tumor-free mice [22], directly linking RNA pol III activity and cancer. Recently, it was demonstrated that BRF1 and TBP are capable of driving oncogenic transformation [16,23]. These observations demonstrate that elevation of RNA pol III transcription contributes to oncogenesis. TFIIIB activity is strictly regulated by Maf1 [24-27], chemopreventative agents [28], and oncogenes and tumor suppressors which are discussed below.

**Figure 2:**
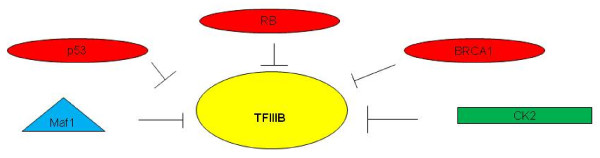
**BRF2-TFIIIB is a target of regulation by tumor suppressors and oncogenic products**. Depiction of tumor suppressor and oncogenic products involved in the regulation of BRF2-TFIIIB. The tumor suppressors, p53, RB and BRCA1 are depicted as red circles. The oncogenic kinase CK2 is depicted by a green box and functions in regulating BRF2-TFIIIB. The stress related protein Maf1 is depicted by a triangle and functions in inhibiting BRF2-TFIIIB.

CK2 (casein kinase 2) is a ubiquitous and conserved protein kinase with growth-promoting and oncogenic properties. CK2 is abnormally active in a variety of human cancers (Figure 2) [29]. It has been demonstrated that CK2 interacts stably with TFIIIB; BRF1 is phosphorylated in cells and CK2 inhibitors can decrease this phosphorylation, thereby promoting transcription complex assembly [29].

TFIIIB activity is targeted by tumor suppressors [30-36] such as ARF [37], RB (Retinoblastoma protein) [30,38], p53 [39-43] and BRCA1 (breast cancer susceptibility gene 1) [44].

RB controls cell growth by preventing cell cycle entry in the absence of appropriate mitogenic signals and inactivation is associated with a variety of human cancers [45]. RB regulates RNA pol III transcription by disrupting interactions between TFIIIB and RNA pol III [38,46-49] RB-mediated repression of U6 transcription can be restored by recombinant SNAPc and TBP [46].

p53 is activated in response to cellular stress, inducing cell cycle arrest or apoptosis, and its inactivation is considered a critical step in carcinogenesis [50]. p53 represses not only Alu and U6 transcription, but also tRNA, 5S rRNA, VAI, B2 and EBER (Epstein-Barr virus) transcription, establishing p53 as a general repressor of RNA pol III transcription [39]. p53 regulates U6 transcription through interaction with the BRF2-TFIIIB subunit TBP [41] and SNAPc [51].

BRCA1 plays a role in DNA repair, cell cycle regulation, apoptosis, genome integrity and ubiquitination [52,53]. Recently, BRCA1 has been characterized as a general repressor of RNA pol III transcription [44]. BRF2 overexpression alleviates BRCA1 mediated repression of U6 transcription [44], suggesting that regulation of U6 transcription by BRCA1 occurs, in part, through BRF2. However, it is currently unclear whether the observed inhibition of RNA pol III transcription is a result of direct or indirect interactions between BRCA1 and BRF2, or BRCA1 and TFIIIB in general.

## BRF2, a general oncogene?

It is established that RNA pol III is often deregulated in cancers [33-35] and specific elevation of RNA pol III transcripts and RNA pol III transcription factors such as U6 snRNA and BRF2 is a feature of both transformed cells and cancers [54]. Recently, Lockwood et al identified BRF2 as a novel oncogene in lung squamous cell carcinoma demonstrating that overexpression of BRF2 can drive expression of RNA pol III transcripts contributing to squamous cell carcinoma tumorigenesis [20]. However, it cannot currently be ruled out that TFIIIB, particularly the BRF2 subunit, could bind and potentially titrate tumor suppressors, thus alleviating some key mechanisms normally keeping TFIIIB activity in check, contributing to oncogenesis. Additionally, no Brf2-dependent pol III transcript has yet been shown to have transforming activity.

RNA pol III is a fundamental determinant of the capacity of a cell to grow and the identification of BRF2 as an oncogene further demonstrates the importance of proper regulation of RNA pol III transcription. Hence, we queried the Oncomine database to systematically assess gene expression levels of BRF2 in a variety of carcinomas. Oncomine is a bioinformatics initiative which collects, standardizes, analyzes, and delivers cancer transcriptome data to the biomedical research community [55]. Rhodes et al. analysis of cancer transcriptome data has identified the genes, pathways, and networks deregulated across 18,000 cancer gene expression microarrays spanning 35 cancer types (for a comprehensive overview of the Oncomine database refer to [55]). Differential expression analysis is an important feature of the Oncomine resource. A unique feature of the Oncomine database is Oncomine automatically computes differential expression profiles for cancer types and subtypes allowing for simple query for individual gene expression.

**Figure 3:**
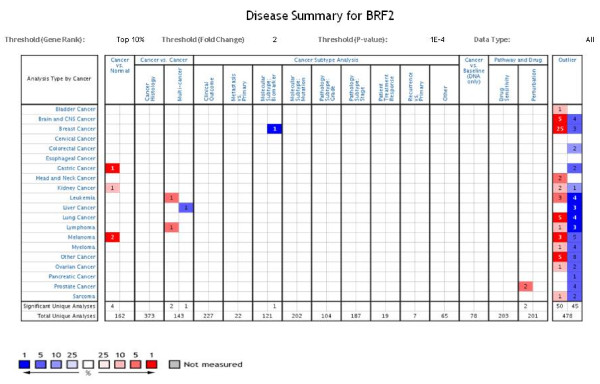
**Oncomine Analysis: Disease Summary for BRF2**. The Oncomine database was queried for BRF2 expression in the available datasets based on the following: cancer type, cancer versus normal, cancer versus cancer, cancer subtype, cancer versus baseline, pathway and drug and outlier analyses. The 'red cells' represents BRF2 overexpression and the 'blue cells' represent BRF2 underexpression. The levels of expression are based on the gene rank percentile. This disease summary was performed using a criterion of a 2 fold change for BRF2 expression and a p-value of 1E-4. Using these stringent criteria for BRF2 expression analysis, we found that BRF2 is highly overexpressed in the following cancer vs normal datasets: gastric, kidney and melanoma cancers. In the cancer vs cancer, multi-cancer datasets, BRF2 is overexpressed in leukemias and lymphomas. The outlier analysis, which is used to determine significant BRF2 expression in a subset of the patient samples, showed that BRF2 is both over- and under-expressed across the analyzed cancers. (Oncomine database).

Thus, using the Oncomine database, we performed a disease summary for BRF2 (Figure 3) to determine if BRF2 overexpression was significant in various carcinomas. Based on this analysis, we determined that in studies comparing cancer versus normal tissue, BRF2 is highly overexpressed in datasets focused on gastric, kidney and melanoma cancers. Please note that sample number for each data set used in our BRF2 analysis is noted, and data sets are named for author whose data set has been analyzed. In Figure 3, overexpression represented by 'red cells' and under expression represented by 'blue cells' is determined based on the gene rank percentile. The outlier analysis, as demonstrated in Figure 3, suggests that there are 50 analyses which have a significant increase in BRF2 expression and 45 analyses which have a significant decrease in BRF2 expression. The outlier analyses represents a small subpopulation of samples within the datasets, hence, they do not reflect the majority of samples. We speculate that out of the 478 unique analyses, the 45 analyses which have a significant decrease of BRF2 expression demonstrates that BRF2 overexpression may not be universal to all cancer patients. The Oncomine database is a compilation of gene expression studies performed from clinically-based analyses performed on patient samples. The 4 analyses which are significant out of 162 that are significant include gastric, kidney and melanoma cancer datasets (Figure 3). This criterion for this specific BRF2 disease summary performed was stringent as we required a p-value of 1E-4 and a fold-change of 2 for BRF2 gene expression compared to the controls. Hence, we believe that due to the stringency of our criterion, only 4 analyses showed a significant increase in BRF2 expression.

**Figure 4:**
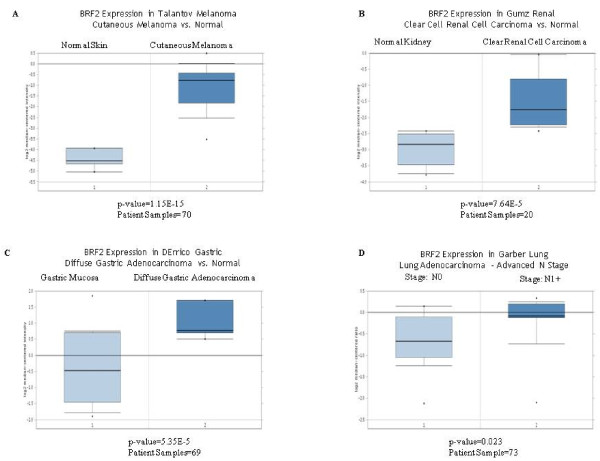
**Oncomine analysis of BRF2 expression in gastric, kidney, melanoma and lung cancers**. As seen in Figure 3, BRF2 is significantly overexpressed in gastric, kidney and melanoma cancer datasets within the Oncomine datasets. We further queried Oncomine for each of these specific cancers to compare the fold change of BRF2 expression in the cancerous tissue compared to the controls for each specific cancer. All p-values represent a student's t-test. Figure 4A shows that BRF2 is overexpressed in cutaneous melanomas compared to normal skin in the Talantov Melanoma dataset [58]. BRF2 overexpression is significant with a p-value of 1.15E-15 and a total of 70 patient samples used for this analysis. Figure 4B shows that BRF2 is overexpressed in clear cell renal carcinoma compared to normal kidney in the Gumz Renal dataset [59]. BRF2 overexpression is significant with a p-value of 7.64E-5 and a total of 20 patient samples were used for this analysis. Figure 4C shows that BRF2 is overexpressed in diffuse gastric adenocarcinoma versus normal gastric mucosa in the DErrico Gastric dataset [60]. BRF2 overexpression is significant with a p-value of 5.35E-5 and a total of 69 patient samples were used for this analysis. Lastly, Figure 4D shows that BRF2 is overexpressed in advance N1+ stage lung adenocarcinoma compared to N0 stage in the Garber Lung Adenocarcinoma dataset [61]. BRF2 overexpression is significant with a p-value of 0.023 and a total of 73 patient samples were analyzed. (Oncomine database).

We further queried the analyses which had a significant increase in BRF2 expression and as demonstrated in Figure 4A, the Talantov Melanoma dataset, comprised of 70 patient samples shows a statistically significant overexpression of BRF2 mRNA, p-value = 1.15E-15, in cutaneous melanoma compared to normal skin. Figure 4B shows a statistically significant overexpression of BRF2 mRNA, p-value = 7.64E-5, in clear renal cell carcinoma compared to normal kidney in the Gumz Renal dataset, comprised of 20 patient samples. Lastly, Figure 4C shows a statistically significant overexpression of BRF2 mRNA, p-value = 5.35E-5, in the DErrico Gastric diffuse gastric adenocarcinoma compared to gastric mucosa dataset, comprised of 69 patient samples. Additionally, Lockwood et al have recently identified BRF2 as an oncogene in lung squamous cell carcinoma [20]. To determine if there was a correlation with BRF2 overexpression and lung carcinoma, we analyzed the Garber Lung dataset, comprised of 73 patient samples, Figure 4D. The data retrieved from the Garber Lung study shows a correlation between BRF2 overexpression and an advanced N stage, N1, in lung carcinoma samples. All statistical values relative to these analyses were calculated as previously described [55].

**Figure 5:**
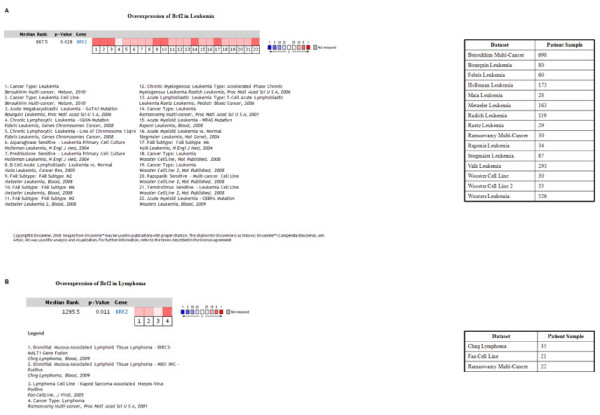
**Oncomine analysis of BRF2 expression in leukemia and lymphoma**. As demonstrated in Figure 3, BRF2 is overexpressed in both leukemia and lymphoma, cancer vs cancer datasets. We further queried the Oncomine database to analyze BRF2 expression in both leukemia and lymphoma. Figure 5A represents an analysis performed across 22 leukemia analyses found within Oncomine. The p-value, 0.028, represents a student's t-test measuring BRF2 overexpression in cancerous tissues, and the red represents significant overexpression. This analysis shows that BRF2 is significantly overexpressed across leukemia. The table states the datasets analyzed and the number of patient samples used in each study. Figure 5B represents an analysis performed across 4 lymphoma analyses found within Oncomine. The p-value, 0.011, represents a student's t-test measuring BRF2 overexpression in cancerous tissue and the red represents significant overexpression. This analysis shows that BRF2 is significantly overexpressed across lymphoma. The table states the datasets used for analysis and the number of patient samples used in each study. (Oncomine database).

Further query of the information presented in the BRF2 disease summary demonstrated that in analyses comparing multi-cancers, "cancer versus cancer", BRF2 is overexpressed in both leukemia and lymphoma as well. In the case of BRF2 expression in leukemia, we searched various leukemia datasets and found that across 22 analyses, BRF2 was significantly overexpressed, Figure 5A, p-value = 0.028. In the case of BRF2 expression in lymphoma, we found that across 4 analyses, BRF2 was significantly overexpressed, Figure 5B, p-value = 0.011.

**Figure 6:**
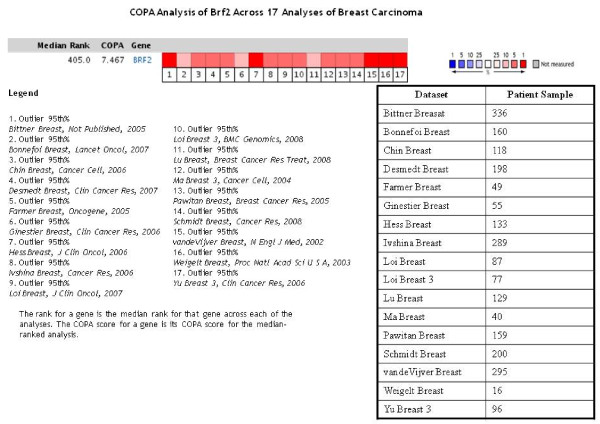
**Oncomine analysis of BRF2 expression in breast carcinoma using COPA**. As seen in Figure 3, BRF2 was overexpressed in a total of 50 analyses using an outlier analysis. COPA (cancer outlier profile analysis) analyzes a small subpopulation of samples which represent outliers within the datasets for significant expression and does not represent the majority of samples. We investigated BRF2 expression in several breast carcinoma studies based on the high number of analyses BRF2 was overexpressed in using COPA (Figure 3). Based on the COPA method, BRF2 is highly overexpressed across 17 breast carcinoma analyses in a subset of the patient samples used for each respective analysis. The red represents significant overexpression. The datasets used for this analysis and the number of patient samples used in each individual study are shown as well. (Oncomine database).

Interestingly, analysis of the BRF2 disease summary shows that BRF2 is highly expressed on the basis of outlier gene expression using a method called COPA (cancer outlier profile analysis). COPA was previously described and utilized to identify oncogenic chromosomal aberrations such as the TMPRSS2:ETS fusion gene in prostate cancer by Tomlins et al [56]. Based on the BRF2 disease summary, we focused on breast carcinoma as this was most significant. Analysis of the 95% outlier across 17 breast carcinoma analyses (Figure 6) shows that BRF2 is highly overexpressed. This demonstrates that in 5% of the samples analyzed in these specific studies, BRF2 overexpression is significant in a small subpopulation of samples.

**Figure 7:**
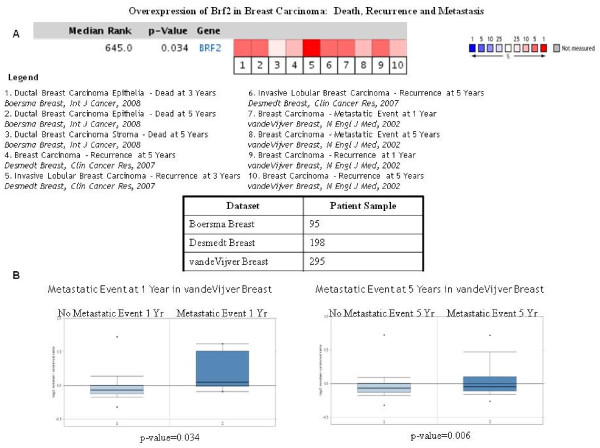
**Oncomine analysis: BRF2 overexpression and clinical outcome**. To determine if BRF2 overexpression correlated with patient outcome in the clinic, we performed further analysis using Oncomine datasets focused on breast carcinoma studies analyzing patient death, recurrence and metastasis. Figure 7A shows that BRF2 is overexpressed across 10 breast carcinoma analyses which focused on patient recurrence at 3 and 5 years; metastasis at 1 and 5 years; and patient death at 3 and 5 years. The p-value, 0.034, represents a student's t-test measuring BRF2 overexpression in the analyzed datasets and the red represents significant overexpression of BRF2. Overall, there is a correlation with BRF2 overexpression and death, recurrence and metastasis in patients diagnosed with breast carcinoma. The datasets used for this analysis and the number of patient samples used in each individual study are shown as well. To further analyze BRF2 overexpression and the correlation with metastasis, we used the vandeVijver breast carcinoma study, (295 patient samples analyzed). Figure 7B demonstrates that in patients presenting with metastasis at 1 and 5 years, there is significant overexpression of BRF2, p-value 0.034 and 0.006 respectively, in comparison to patients with no metastasis. (Oncomine database).

Lastly, we investigated if there was a correlation between BRF2 overexpression and clinical outcome. Using breast carcinoma studies, we performed a meta-analysis across 10 different breast carcinoma datasets studying recurrence, metastasis and death, Figure 7A. We determined that BRF2 overexpression did indeed correlate with clinical outcome. Figure 7B represents one dataset analyzed in Figure 7A, the vandeVijver breast carcinoma study, and shows that BRF2 overexpression is highly significant in patients presenting with metastasis at year 1, p-value = 0.034, and at year 5, p-value = 0.006. This data set is extremely significant at it shows promise for the potential use of BRF2 as a potential biomarker for patients at risk for metastasis and may serve as a potential therapeutic target.

Recently, RNA pol III transcription has been the focus of a Phase I and pharmacokinetic study. Hammond-Thelin et al. studied the effects of a novel nucleoside analog inhibitor (TAS-106) of RNA pol I, II and III, in patients with advanced solid malignancies [57]. Previously, TAS-106 has demonstrated antitumor activity in various human cancer models including leukemic, lung, colorectal, stomach, pancreatic, and gastric cancers [57]. The principal objectives of the study were to determine the maximum tolerated dose in patients, characterize the toxicities associated with TAS-106 administration, determine the pharmacokinetics of TAS-106 and study if there was any indication of antitumor activity in patients [57]. Although this study is in its infancy, it's representative of the potential use of RNA pol III inhibitors as a means of pharmacological target for the treatment of cancers.

## Conclusions

By elucidating the mechanism(s) by which RNA pol III transcription is both regulated and deregulated, it will be possible to further understand the mechanism(s) by which aberrant activity of the general transcription machinery contributes to cancer development. Deregulation of RNA pol III transcription in cancers coupled with the observation that TFIIIB, specifically BRF2-TFIIIB, is commonly a target of deregulation in a variety of cancers demonstrates that RNA pol III transcription is indeed a key player in tumorigenesis and could serve as a novel target in the development of pharmacological agents.

## Competing interests

The authors declare that they have no competing interests.

## Authors' contributions

SC reviewed the literature, wrote and drafted the manuscript; LS corrected and finalized the manuscript. All authors read and approved the final version.
